# A qualitative examination of home and neighborhood environments for obesity prevention in rural adults

**DOI:** 10.1186/1479-5868-5-65

**Published:** 2008-12-10

**Authors:** Michelle C Kegler, Cam Escoffery, Iris Alcantara, Denise Ballard, Karen Glanz

**Affiliations:** 1 Department of Behavioral Sciences and Health Education, Emory Prevention Research Center, Rollins School of Public Health, Emory University, Atlanta, Georgia, USA; 2 Department of Behavioral Sciences and Health Education, Rollins School of Public Health, Emory University, Atlanta, Georgia, USA; 3 Emory Prevention Research Center, Rollins School of Public Health, Emory University, Atlanta, Georgia, USA; 4 Southwest Georgia Cancer Coalition, Albany, Georgia, USA; 5 Department of Behavioral Sciences and Health Education, Emory Prevention Research Center, Rollins School of Public Health, Emory University, Atlanta, Georgia, USA

## Abstract

**Background:**

The home and neighborhood environments may be important in obesity prevention by virtue of food availability, food preparation, cues and opportunities for physical activity, and family support. To date, little research has examined how home and neighborhood environments in rural communities may support or hinder healthy eating and physical activity. This paper reports characteristics of rural homes and neighborhoods related to physical activity environments, availability of healthy foods, and family support for physical activity and maintaining an ideal body weight.

**Methods:**

In-depth interviews were conducted with 60 African American and White adults over 50 years of age in two rural counties in Southwest Georgia. Interviews were transcribed verbatim and coded independently by two members of the research team using standard methods of qualitative analysis. Themes were then identified and data matrices were used to identify patterns by gender or race.

**Results:**

Neighborhood features that supported physical activity were plenty of land, minimal traffic and living in a safe and friendly neighborhood. The major barrier was lack of recreational facilities. The majority of participants were not physically active with their family members due to schedule conflicts and lack of time. Family member-initiated efforts to encourage physical activity met with mixed results, with refusals, procrastination, and increased activity all reported. Participants generally reported it was easy to get healthy foods, although cost barriers and the need to drive to a larger town for a supermarket with good variety were noted as obstacles. Family conversations about weight had occurred for about half of the participants, with reactions ranging from agreement about the need to lose weight to frustration.

**Conclusion:**

This study suggests that successful environmental change strategies to promote physical activity and healthy eating in rural neighborhoods may differ from those used in urban neighborhoods. The findings also provide insight into the complexities of family support for physical activity and maintaining a healthy weight. Addressing socio-ecologic factors has the potential to increase healthy behaviors and decrease the prevalence of obesity among rural residents.

## Background

The prevalence of overweight and obesity in the United States has increased markedly in the past decade [1]. In 2005–2006, 33% of men and 35% of women were obese [2]. Excess weight is associated with the development of type 2 diabetes, cardiovascular disease, some types of cancer, and other chronic conditions [3]. Obesity is more common among rural residents than urban residents. Prevalence estimates based on self-reported height and weight suggest rural residents are 12–15% more likely to be obese than urban residents [4-6]. Rural residents are also less likely to be physically active than their urban counterparts, particularly in the South [6-9].

The major behavioral contributors to overweight and obesity are excess food consumption and inadequate physical activity [3]. Although eating and activity are individual behaviors, growing evidence suggests that the problem of obesity is powerfully influenced by social and built environments [10,11]. Research on neighborhood walkability in urban areas, for example, suggests that people are more physically active when they live in neighborhoods with higher residential density, a mixture of land uses, recreational facilities, connected streets, and enjoyable scenery [12-15]. In contrast, rural areas are characterized by lower residential density, less varied land use, fewer sidewalks, fewer streetlights and limited access to recreational facilities [8,16,17]. As a result, walking for transport may be less common in rural areas and having fewer environmental supports may increase the relative importance of social and individual determinants of physical activity. The decline of downtown shopping districts in small rural towns and the automation of agricultural, livestock and logging industries may also contribute to less active lifestyles [18]. Research has documented that not seeing others exercise in the neighborhood, not having enjoyable scenery, feeling unsafe from crime and distance to nearest recreational facility are associated with sedentary behavior and obesity among rural adults [8,16].

Home and neighborhood nutrition environments are also important in obesity prevention by virtue of the greater availability of some foods than others, shared food preparation and meal patterns [19,20]. To date, research on home nutrition environments has focused largely on youth, with minimal attention paid to how home nutrition environments may differ by neighborhood or how food available in the home may affect adult eating patterns [21]. More attention has been paid to neighborhood nutrition environments such as the accessibility of supermarkets which are less common in rural areas [22-25]. Given that shopping at supermarkets is associated with greater fruit and vegetable consumption and traveling greater distances to grocery shop is associated with higher body mass index, the scarcity of supermarkets in rural areas may help explain the increased rates of obesity [26,27].

Social environments also impact behaviors. Family social support, including encouragement or sabotage from household members, is associated with dietary behaviors and physical activity in some studies, although findings are inconsistent across studies [28-30]. In a study on women and physical activity, rural women were more likely than urban women to say that others discouraged them from exercising and that caregiving duties prevented them from exercising [8]. In addition to providing social support, family members can serve as proximal leverage points for changing the home environment [31]. The family members who shop for groceries and prepare the food, for example, can shape the home nutrition environment through availability and access to certain kinds of foods in the home [21,32].

The purpose of this qualitative study was to provide an in-depth understanding of how the home and neighborhood environment may affect healthy eating and physical activity in rural communities from the perspective of older adults. We examined aspects of both the social and built environments, specifically characteristics of rural neighborhoods that support or hinder physical activity, availability of healthy foods in the home and neighborhood, and family support for physical activity and maintaining an ideal body weight. Data are from a larger qualitative study that examined how rural home, worksite and church environments may influence tobacco use, physical activity and healthy eating.

## Methods

This research was conducted through a partnership between the Emory Prevention Research Center (EPRC), the Southwest Georgia Cancer Coalition and a Community Advisory Board (CAB) of representatives from multiple sectors. In keeping with community-based participatory research [33], the CAB made major decisions about study design such as eligibility criteria, helped develop the data collection instrument, and aided in interpreting results. Data collection was supervised by Southwest Georgia Cancer Coalition staff and local residents were hired to conduct the interviews. The research protocol was reviewed and approved by the Emory University Institutional Review Board.

### Study Participants

Eligible study participants were African American or White, over 50 years of age, lived with at least one other person and had resided in the contiguous counties of Terrell County or Calhoun County, Georgia for at least ten years. Calhoun (population 6,094) and Terrell (population 10,657) Counties are located in rural Southwest Georgia and are similarly characterized by poverty and low educational attainment [34]. In both counties, approximately 35% of adults 25 years of age and older have not earned a high school diploma and 23% of families live below the federal poverty level. Both counties have large African American populations (> 60% of residents), with significant disparities in income and education levels between African American and White residents. A purposive sampling approach was used to recruit 60 participants evenly divided by gender and race. The CAB chose to focus on adults aged 50 and older because the research findings would be used to inform the development of an intervention to prevent cancer and members believed that prevention of cancer and other chronic diseases would be more salient at this life stage.

### Data Collection Procedures

Participants were recruited by the interviewers through snowball sampling using personal contacts and local businesses and organizations as starting points. The data were collected using semi-structured interviews in participants' homes in 2005. All participants provided written informed consent. The interviews were conducted by race- and gender-matched local residents. Interviewers attended a 1.5 day training in qualitative interview methods and completed several pilot interviews.

The interview guide was developed collaboratively with CAB members to address their priorities for understanding primary prevention of cancer and to address gaps in the literature on how rural social and built environments may influence physical activity, healthy eating and tobacco use. The interview guide was pilot tested by CAB members and revised based on their feedback. The interviews typically took 60 minutes to complete and covered how social and physical environments in the home, work and church influence healthy eating, physical activity and tobacco use. Table 1 lists questions asked about the home environment, physical activity and healthy eating included in the analyses reported here.

**Table 1:** Interview Questions on Home Environment, Healthy Eating and Physical Activity

**Topic**	**Question & Probes**
Neighborhood Features that Support or Hinder Physical Activity	What, if anything, about your neighborhood or nearby community makes it easy to be physically active. Probe on: walking trails, parks, streetlights, sidewalks, etc.
	What, if anything, about your neighborhood or nearby community makes it hard to be physically active. Probe on: broken or no sidewalks, crime, loose dogs, traffic, etc.

Availability of Healthy Foods	Would you say it is easy or hard to get healthy foods for your family or household? What are some reasons for that?
	How often do you have fresh fruits and vegetables available in your home?
	Does your family or household have a vegetable garden? How often do you eat fresh or canned foods from your garden?

Family Support for Ideal Body Weight and/or Weight Loss	Has anyone in your family or household ever talked to you about your weight? Who brought it up? What did they say? Did it make you want to try to lose weight? How did you react?

Family Support for Physical Activity	How often, if at all, do you and your family or household members participate in physical activities together? What do you do? What do you think are some reasons for that [not being active together]?
	Have you or anyone else tried to encourage your family or household to be more physically active together? What happened with that? Who brought it up? Did it work? How did people react?
	What could your family or household do to encourage you to be more physically active?

### Data Analysis

The interviews were transcribed verbatim. A codebook was developed to capture major themes for each topic covered in the interviews. Two coders then coded each transcript independently, with discrepancies resolved through consensus. The QSR-N6 software was used for data storage, retrieval, and analysis [35]. Reports which contained all comments associated with particular codes or combinations of codes were generated using N6. Conventional content analysis was performed to identify the full range of responses [36]. Matrices were then constructed to aid in identifying patterns and themes by gender and race [37,38]. For example, one of the matrices for the "family conversations about weight" listed all responses related to content of these conversations by race and gender. The cases associated with each response were referenced in the cells. A response or set of responses was labeled as a theme if multiple participants (≥ 5) discussed it. This approach allowed us to assess the strength of theme and possible patterns by race and gender. It also provided an audit trail to increase trustworthiness of the findings. Themes, as well as the full range of responses, are reported in the results. Matrices were also used to explore patterns between categories of codes, for example, between neighborhood features that support or inhibit physical activity and family-based physical activity and between availability of fruits and vegetables in the home and grocery shopping practices.

## Results

### Description of Study Participants

Participants were 48.3% female and 51.7% African American (Table 2). Mean age was 62.8 years, 75.9% were married or living with a partner, and 39.7% lived in a household with at least two other persons. A relatively large percentage (41.4%) of participants reported less than a high school education and 15.5% reported an annual household income of less than $10,000.

**Table 2:** Description of Study Participants

**Characteristic^1^**	**Total^2^** **N = 58**	**African American** **N = 30**	**White** **N = 28**
Gender, n(%)			
Male	30 (51.7)	15 (50.0)	15 (53.6)
Female	28 (48.3)	15 (50.0)	13 (46.4)

Average Age, in years	62.8	62.3	63.5
Range	51–84	51–81	51–84

Education, n(%)			
Less than HS	24 (41.4)	22 (73.4)	2 (7.1)
HS graduate	7 (12.1)	3 (10.0)	4 (14.3)
Some college/vo-tech	12 (20.7)	1 (3.3)	11 (39.3)
College graduate	7 (12.1)	1 (3.3)	6 (21.5)
Graduate degree	7 (12.1)	3 (10.0)	4 (14.3)

Household Income, n(%)			
$10,000 or less	9 (15.5)	8 (26.7)	1 (3.6)
$10,001 to $25,000	12 (20.7)	10 (33.3)	2 (7.1)
$25,001 to $50,000	7 (12.1)	4 (13.3)	3 (10.7)
$50,001 or more	20 (34.5)	2 (6.7)	18 (64.3)
*Refused*	10 (17.2)	6 (20.0)	4 (14.3)

Marital Status, n(%)			
Married	42 (72.4)	17 (56.7)	25 (89.3)
Living with someone	2 (3.4)	2 (6.7)	0 (0)
Divorced or separated	5 (8.6)	5 (16.7)	0 (0)
Single	2 (3.4)	2 (6.7)	0 (0)
Widowed	6 (10.3)	4 (13.3)	2 (7.1)

Household Size, n(%)			
2	34 (58.6)	15 (50.0)	19 (67.9)
3	11 (19.0)	6 (20.0)	5 (17.9)
4	7 (12.1)	4 (13.3)	3 (10.7)
5 or more	5 (8.6)	5 (16.7)	0 (0)

County, n(%)			
Calhoun	13 (22.4)	4 (13.3)	9 (32.1)
Terrell	45 (77.6)	26 (86.7)	19 (67.9)

^1 ^Percentages are out of total respondents. In cases of missing data, totals do not equal 100%

^2 ^Two interviews were not successfully tape-recorded so complete data are available on 58 of 60 participants

### Neighborhood Features that Support Physical Activity

Participants were asked what factors in their neighborhoods or nearby communities made it easy to be physically active. Themes included plenty of space for walking and riding bikes, particularly up and down the road outside their homes; minimal traffic; and living in a safe and friendly neighborhood. Each of these is illustrated below:

Plenty of Space for Physical Activity

*I live in the country, and I have all the room in the world to walk, get around*. (White Female)

Minimal Traffic

*It's in a rural area, so we can get out and walk on the street and not be worried about vehicles because we don't have them, the traffic is very slow*. (White Female)

Safe and Friendly Neighborhood

*We're a small town and everybody knows each other and it's safe to get out and walk and you have lots of friends that will walk with you*. (White Male)

When asked specifically about the presence of sidewalks and streetlights, participants generally commented on whether they existed or not, but typically did not elaborate on whether sidewalks and lighting facilitated physical activity. When asked directly about the availability of recreation facilities, tracks and walking trails were mentioned most often.

### Neighborhood Characteristics that Hinder Physical Activity

Participants were also asked what factors in their neighborhoods or nearby communities make it hard to be physically active. A major theme was lack of access to exercise or recreational facilities.

Lack of Recreation Facilities

*There are no walking trails or anything. There are sidewalks [...]. There are no gyms, no, really, it's a small town. There's really nothing. [...] There's not a track or anything like that. So, you're basically walking on, I hesitate to use the word city with a small town, but you're walking in town, town streets, and crossing streets, and traffic and all*. (White Female)

When asked specifically about lack of sidewalks and streetlights, quite a few reported not having sidewalks. Lack of streetlights appeared less common, but was mentioned by a few, primarily as a deterrent to exercising after dark. Additional barriers to physical activity within specific neighborhoods included: loose dogs, heavy or speeding traffic, and crime or concerns about safety.

Loose Dogs

*I have to wait 'til somebody come and walk with me 'cause I'm scared of them dogs. When they get at me and they run (laughing)*. (African-American Female)

Heavy or Speeding Traffic

*...they don't have no compassion for a person walking. They're concerned, you see me coming, you better get out of the road*. (African-American Male)

Concerns over Crime and/or Safety

*I used to walk in the mornings, I did so for a good little while with no problem, and then in the path that I walked there was a person that all of a sudden showed up and was sitting in a certain area, and it was very uncomfortable, because it was not something that I had been seeing normally. So, that stopped me from walking by myself in that area*. (White Female)

A final theme was that nothing about the neighborhood made it difficult to be physically active.

### Family Support for Physical Activity

We were also interested in family support for physical activity. Participants were asked how often, if at all, their family participates in physical activities together. The majority stated their families were not physically active together. The two most commonly cited reasons for not being physically active together were schedule conflicts and the lack of time and opportunity.

Schedule Conflicts

*...we don't 'cause everybody here and there so we don't 'cause my husband used to walk before he went to work and it be so early I still be you know and I walk after daylight, so it's a long [time] since we walked together*. (African-American Female)

Lack of Time

*Well one of the reasons, we don't have as much time to do it and we so tied up doing other things that we don't, we won't take the time to do it. I won't say we don't have it but we can't make the time for more*. (African-American Male)

Additional reasons for families not engaging in physical activity together include: laziness, less motivation now that children are grown, differing exercise preferences including walking pace, spouses refusing even when asked, physical limitations and caretaking responsibilities.

Among participants who stated their family *does *participate in physical activities together, taking walks was the most common form of exercise, followed by sports such as tennis and basketball, biking, yard work, and swimming. Lifting weights, dancing and going to a rehabilitation center were also mentioned.

When asked if s/he or anyone in the family had *ever tried *to encourage the family to be more physically active together, most participants responded affirmatively. In addition to themselves or their spouses encouraging physical activity, several mentioned that their children had made efforts to get the family to be more physically active.

Family efforts to encourage physical activity included: inviting a family member to take a walk, talking about physical activity, or in a few instances, purchasing equipment. In families where someone did encourage the family to be more physically active together, about half said such efforts were not terribly effective. Two common responses were outright refusals or procrastination. Others discussed schedule conflicts and shortness of breath due to smoking as making it difficult for family members to be physically active together.

Refuse to Engage in Physical Activity with Family Members

*Uh huh. But he said he's not walking for nobody and he don't do .... Played a lot of ball, sports, and stuff, but he don't do any of that now*. (African-American Female)

Procrastinate

*We've talked about it, and we know that we need to, but we just hadn't gotten, I guess you can say we're procrastinating*. (White Female)

A smaller number reported that efforts did produce positive results, with families walking, playing golf, riding bikes, fishing, and relieving stress through physical activity.

When asked what their family or household could do to encourage them to be more physically active, about half of the interviewees responded that their family could not do anything. These participants explained they were already active or were doing well for their age with existing practices. Another theme was that being physically active depended on the individual, not the family or anyone else.

Among those who stated their family could do something to encourage them to be more physically active, participation in or invitations to engage in physical activities were viewed as helpful. Verbal support or encouragement would also help, as would taking the time or committing to be physically active together.

We were also interested in how features of the neighborhood might affect whether families engage in physical activity together. Interestingly, those that described their neighborhoods as having several of the features that typically support walking (e.g., sidewalks, safe, limited traffic) generally did not engage in family-based physical activity, similar to the rest of the participants. Moreover, participants who reported that someone in their family had at least encouraged family-based physical activity did not necessarily live in the more walkable neighborhoods.

### Availability of Healthy Foods in the Community

When asked whether it was easy or hard to get healthy foods for the family, most indicated it was easy. When asked to explain why they thought it was easy, the two strongest themes were that it was easy to get healthy foods at most grocery stores and that healthy food is generally affordable. Only a couple of respondents highlighted how living in a rural area made it especially easy to get healthy foods.

Easy to Get Healthy Foods at Grocery Stores

*It's easy, 'cause you can always go to the store and get yourself a veggie*. (African-American Female)

Healthy Foods are Affordable

*If you want to get it, because you don't pay no more or less to go down there and buy cabbage, rutabaga, squash, than you buy this other stuff. No, it's not hard, it's just a matter of wanting to do that. It's a choice, it's my choice*. (African-American Female)

Easy to Get Healthy Foods in Rural Communities

*I don't think it's hard to get healthy foods, especially in an area where, a rural area where a lot of farming go, it easy to get fresh vegetables. It's easy to get fruit or, you know, than less healthy foods*. (African-American Male)

Among those who expressed difficulty in getting healthy foods for the family, the cost of healthy foods was cited as the main barrier. Other barriers included poor selection at local stores, limited time to shop and the price of gas. A few participants spoke of driving to larger communities with bigger grocery stores in order to get a better selection of healthy foods. Weekly grocery shopping trips were most common by far, followed by 2–3 trips per month. Small trips (e.g., picking up a few items) between big grocery shopping trips were also common. About half described driving 15 to 45 miles for some of their grocery shopping. Others almost always shopped at the smaller stores in their own towns.

### Availability of Healthy Foods in the Home

Study participants were asked about the availability of fruits and/or vegetables in their home. The majority reported they were frequently available. Of those reporting having fruit and vegetables available in the home sometimes, over half reported shopping at least weekly.

Frequently Available

*All the time, it's available. We don't cook it every night, on the occasions we cook we a lot of time we'll have fresh fruit or vegetables and then we have usually fresh fruit, we'll have strawberries or peaches or something a lot of times in the morning on cereal and then at night we'll sometimes just have some fruit. We usually keep some sort of fresh fruit and vegetables in the house. Eat a lot of raw carrots*. (White Male)

Sometimes Available

*Because of the price of them, we don't, they might, it might be, they might be some and it might not be, you know. I like apples and bananas, and what not, and grapes and all that, but we don't eat them all the time*. (African-American Male)

About half of interviewees reported that they also frequently have junk food available at home.

### Presence of Family Vegetable Gardens

Participants were also asked if their family or household had a vegetable garden. Most indicated they did not have a produce garden or regular access to one. Reasons included having no time, being too old, being able to buy produce cheaper than actually growing it, and that gardens were too much work.

Too Old and No Time

*No, too old for a garden. One time we had one, a long time ago. But that's long past. My husband had one but I don't have time for a vegetable garden. But my neighbor has one*. (African-American Female)

Too Much Work and Cheaper to Buy Produce

*Shoot man that's too much work, I can buy it cheaper than I can raise it*. (African-American Male)

### Family Support for Maintaining an Ideal Body Weight

When asked if anyone in their family has talked about their weight or maintaining an ideal body weight, about half of the interviewees indicated that they have had discussions with family members, most often a spouse. Of those who gave details about the content of their conversations, most indicated that a family member usually mentions his or her weight gain by expressing concern, or stating that s/he is getting too fat or too heavy. A few indicated that a family member had explicitly suggested he or she lose weight or offered to help them lose weight. A few participants also reported being teased by a family member regarding their weight.

Family Members Mention Weight Gain

*Well my husband talked about it and he would say you don't look like the same woman I married...you so heavy, this and that... *(African-American Female)

Family Members Encourage Weight Loss

*Other than she's been concerned with my weight just as much as I was, and she's helped me when I wanted to lose weight, I started on weight loss programs... *(White Male)

Family Members Tease About Weight

*They joke about my weight, until I stop laughing, and then the joke stop*. (African-American Male)

Participants who described conversations with family members about their weight were also prompted to discuss their reactions to these conversations. Participants generally affirmed wanting to lose weight, although another common reaction among women was to get angry or frustrated. A couple of female participants were not bothered by their spouse's comments on their weight.

Desire to Lose Weight

*Oh, I say, oh I'm going to lose this weight, I'm going to lose this weight, shut up*. (African-American Male)

Frustrated by Comments

*Frustrated, because I know he's right, and I really just don't want to hear about it*. (White Female)

Not Bothered Anymore

*I used to try to do it when he say something, but now I don't, it don't bother me no more 'cause now he's big (laughing)*. (African-American Female)

About half said that there had NOT been any type of discussion of body weight in their families. The most common explanations were that their weight has never really been a problem or that weight is a personal issue. Interestingly, more participants reported discussing physical activity with their families than weight issues. Some families had discussed both weight and physical activity, some had discussed one or the other, and some had not discussed either topic.

## Discussion

This study adds to the understanding of family and neighborhood determinants of healthy eating and physical activity by focusing on older adults living in rural communities. Moreover, the qualitative nature of the study provides insight into findings from previous research, such as limited associations between physical activity and environmental variables among rural residents, and equivocal findings on the role of family support in physical activity and healthy eating.

Most research on neighborhood walkability has been conducted in urban areas and supports the importance of higher residential density, mixed use and connected streets as facilitators for walking [12-15]. Our participants, in contrast, emphasized plenty of space for walking, in addition to minimal traffic and living in safe and friendly neighborhoods. Given the lack of mixed use development in rural areas, and the associated scarcity of destinations such as stores or restaurants within walking distance, neighborhood characteristics that support walking likely differ between urban and rural locations [18]. In addition, the paucity of features typically associated with walking (e.g., fewer recreational facilities, limited sidewalks), may help explain why physical activity rates are lower among rural residents, at least in the southeastern U.S. Our findings on the barriers to physical activity were similar to findings on barriers reported by others [8,39].

This study also explored the perceived availability of healthy foods in both the home and community environment. Although most participants reported that they often or frequently had fruits and vegetables available in their homes, the cost of healthy foods and access to larger grocery stores for a wider selection emerged as potential barriers for some rural residents. Other studies have documented that rural residents live a greater distance from larger supermarkets and often have more direct access to convenience stores that typically have higher prices for the more nutritious options [24,25]. A significant number of our participants reported driving a considerable distance to grocery shop for at least some of their trips. Frequency of shopping at larger stores with better selection and lower-priced healthy foods may affect how often fruits and vegetables are available in the home environment.

This study also explored the role of family social support in physical activity and weight management. Our findings corroborate the association between physical activity and family support noted in other studies [8,30,39,40]. The majority of participants reported that their families were not physically active together, but that family members, often spouses or children, did play a role in encouraging physical activity. Results of these efforts were mixed, however, with refusal or procrastination by some family members and increased participation in physical activity by others. Neighborhood walkability did not appear to influence whether families exercised together or whether someone in the family had encouraged physical activity.

Prior research shows that family members can be supportive in improving dietary behaviors by encouraging change, and that individuals turn to spouses or other family members as primary support for improving diet and activity [28,41]. In our study, about half of participants reported discussions with family members about their weight. Anger and frustration were common reactions, particularly among women, to a family member's encouragement to lose weight. This suggests that family social support may be complex and vary by gender or other family dynamics. Our findings also indicated that family conversations about weight loss do not consistently include physical activity.

This study has several limitations. First, our participants were older African American and white residents of rural, low-income counties in the southeastern U.S., with 41.4% reporting less than a high school education. As a result, our findings are not transferable to other types of rural communities. Second, although all participants lived in rural counties, they lived in a variety of types of neighborhoods. For example, some lived on farms with few nearby neighbors and others lived in neighborhoods within small towns. It is likely that neighborhood determinants of physical activity and nutrition vary for these different types of rural neighborhoods. Third, the possibility of socially desirable responses also exists, with participants potentially exaggerating family and neighborhood support for healthy behaviors.

This study has numerous implications for health promotion practitioners working in rural communities. Our study, as well as past research, suggests that the distance to recreational facilities is a particularly common barrier to physical activity for rural residents [42,43]. Establishing walking trails is a relatively low-cost approach to removing some of the environmental barriers found in this study and walking trails have been shown to increase amount of time walking [42]. In addition, the positive aspects of rural areas, such as space for walking and minimal traffic, should be emphasized, and the use of existing low-cost or free facilities (e.g, high school fields or tracks) should be encouraged. Our study found that most participants did not have a produce garden or access to one. Berti and colleagues reported that home gardens had a higher success rate in increasing nutrition outcomes than other strategies in a review of agricultural interventions [44]. Although promoting gardening could serve two purposes, increasing access to fruit and vegetables and increasing physical activity, barriers such as cost and skills would need to be addressed.

Our study also suggests several areas for future research. It is unclear, for example, whether urban findings, such as mixed use development, connected streets and sidewalks, etc, apply to walking behavior in rural areas. Eyler et al. found that traffic, sidewalks, safety and destinations within walking distance were not associated with physical activity among rural women in the Midwest [45]; Sanderson reported parallel findings for rural African American women [46]. Similar questions arise for healthy eating. How does the scarcity of large supermarkets in small towns affect fruit and vegetable consumption? Is it moderated by the availability of local produce through other mechanisms, or by weekly shopping trips into a larger town? Based on our study findings, family social support appears to have potential, but is not uniformly effective. Under what circumstances is family social support helpful and when is it detrimental? Are certain types of support (i.e., emotional, informational) more effective than others in promoting healthy eating and physical activity? Does it vary by gender? Lastly, additional intervention research is needed to develop and evaluate strategies to increase healthy eating and physical activity among rural families.

## Competing interests

The authors declare that they have no competing interests.

## Authors' contributions

MK led the design and implementation of the study, directed data analysis, drafted most of the methods and results sections and edited the entire manuscript. CE contributed to the design and implementation of the study, and drafted part of the discussion section. IA participated in data analysis, and drafted parts of the introduction and results sections. DB helped to interpret findings and drafted part of the discussion section. KG contributed to study design and drafted part of the introduction. All authors edited the entire manuscript, and read and approved the final manuscript.
